# Dynamic transcriptomic changes of goat abomasal mucosa in response to *Haemonchus contortus* infection

**DOI:** 10.1186/s13567-020-00768-y

**Published:** 2020-03-16

**Authors:** Hadeer M. Aboshady, Nathalie Mandonnet, Yoann Félicité, Julien Hira, Aurore Fourcot, Claude Barbier, Anna M. Johansson, Elisabeth Jonas, Jean-Christophe Bambou

**Affiliations:** 1 AgroParisTech, Paris, France; 2 Department of Animal Breeding and Genetics, Swedish University of Agriculture Science, Uppsala, Sweden; 3 URZ Recherches Zootechniques, INRAE, 97170 Petit-Bourg, Guadeloupe France; 4 Department of Animal Production, Faculty of Agriculture, Cairo University, Cairo, Egypt; 5 UEPTEA Plateforme Tropicale d’Expérimentation sur l’Animal, INRAE, 97170 Petit-Bourg, Guadeloupe France

## Abstract

Gastrointestinal nematode (GIN) infections are one of the major constraints for grazing sheep and goat production worldwide. Genetic selection for resistant animals is a promising control strategy. Whole-transcriptome analysis via RNA-sequencing (RNA-seq) provides knowledge of the mechanisms responsible for complex traits such as resistance to GIN infections. In this study, we used RNA-seq to monitor the dynamics of the response of the abomasal mucosa of Creole goat kids infected with *Haemonchus contortus* by comparing resistant and susceptible genotypes. A total of 8 cannulated kids, 4 susceptible and 4 resistant to GIN, were infected twice with 10 000 L3 *H. contortus*. During the second infection, abomasal mucosal biopsies were collected at 0, 8, 15 and 35 days post-infection (dpi) from all kids for RNA-seq analysis. The resistant animals showed early activation of biological processes related to the immune response. The top 20 canonical pathways of differentially expressed genes for different comparison showed activation of the immune response through many relevant pathways including the Th1 response. Interestingly, our results showed a simultaneous time series activation of Th2 related genes in resistant compared to susceptible kids.

## Introduction

Gastrointestinal nematodes (GIN) are an important constraint on grazing ruminants worldwide. These parasites can cause mortality especially in small ruminants but their main effect is reduced productivity [1, 2]. Anthelmintic treatments are the mainstay of current treatment but are threatened by the evolution of drug resistance in parasite populations [3]. Besides, the environmental side-effect of anthelmintic residues is no longer desirable for sustainable production and the increased demand for chemical-free animal products. Therefore, there is a need for additional control strategies. The introduction of resistance to GIN traits in small ruminants breeding schemes, would be a promising sustainable method to control GIN infection [1, 4, 5].

Resistance against most of the common diseases are complex traits involving many genes, the detection of causative variations is therefore a complex task. Currently selection against GIN relies on indirect measures such as fecal egg count (FEC), packed cell volume (PCV) and blood eosinophilia [6–9]. A major disadvantage of these methods is that animals must be infected either naturally or experimentally for these measures. An alternative is the identification and the selection of genes that are responsible for resistance to GIN infection. Several studies investigated the molecular and cellular processes associated with GIN resistance in different tissues such as duodenal [10–12] and abomasal mucosa [13, 14] and draining lymph nodes [15–19] mainly in sheep. However, only a few studies have investigated the biological processes associated with GIN resistance in goats.

It has been shown that whole-transcriptome analysis via RNA-seq is a key tool to investigate the molecular mechanisms responsible for complex quantitative traits such as resistance to GIN infection [20]. A detailed understanding of the genes and biological mechanisms involved in resistance and protective immunity would provide new phenotypic and genetic markers for effective breeding schemes [21].

Previously, we investigated the transcriptome variation in response to GIN infection in goats at 42 days post-infection (dpi) [22]. The results indicated that the maintenance of the integrity of the mucosa was probably the priority for the host at this late infection stage (42 dpi). The present study aimed to identify the changes over time in the molecular pathways and immunity development in response of Creole goats to GIN infection by analyzing the transcriptome of abomasal mucosa of resistant and susceptible kids at different time points post-infection.

## Materials and methods

### Ethics statement

All animal care handling techniques and procedures as well as the procedures for experimental infection, tissue sampling and slaughtering were approved by the French Ethic Committee n°069 (Comité d’Ethique en Matière d’Expérimentation Animale des Antilles et de la Guyane, CEMEAAG) authorized by the French Ministry of Higher Education, Research and Innovation. The experiment was performed at the INRA Experimental Facilities PTEA (Plateforme Tropicale d’Expérimentation sur l’Animal), in Guadeloupe (French West Indies) (16° 20′ latitude North, 61° 30′ longitude West), according to the certificate number A 971-18-02 of authorization to experiment on living animals issued by the French Ministry of Agriculture.

### Animals and experimental design

**Figure 1:**
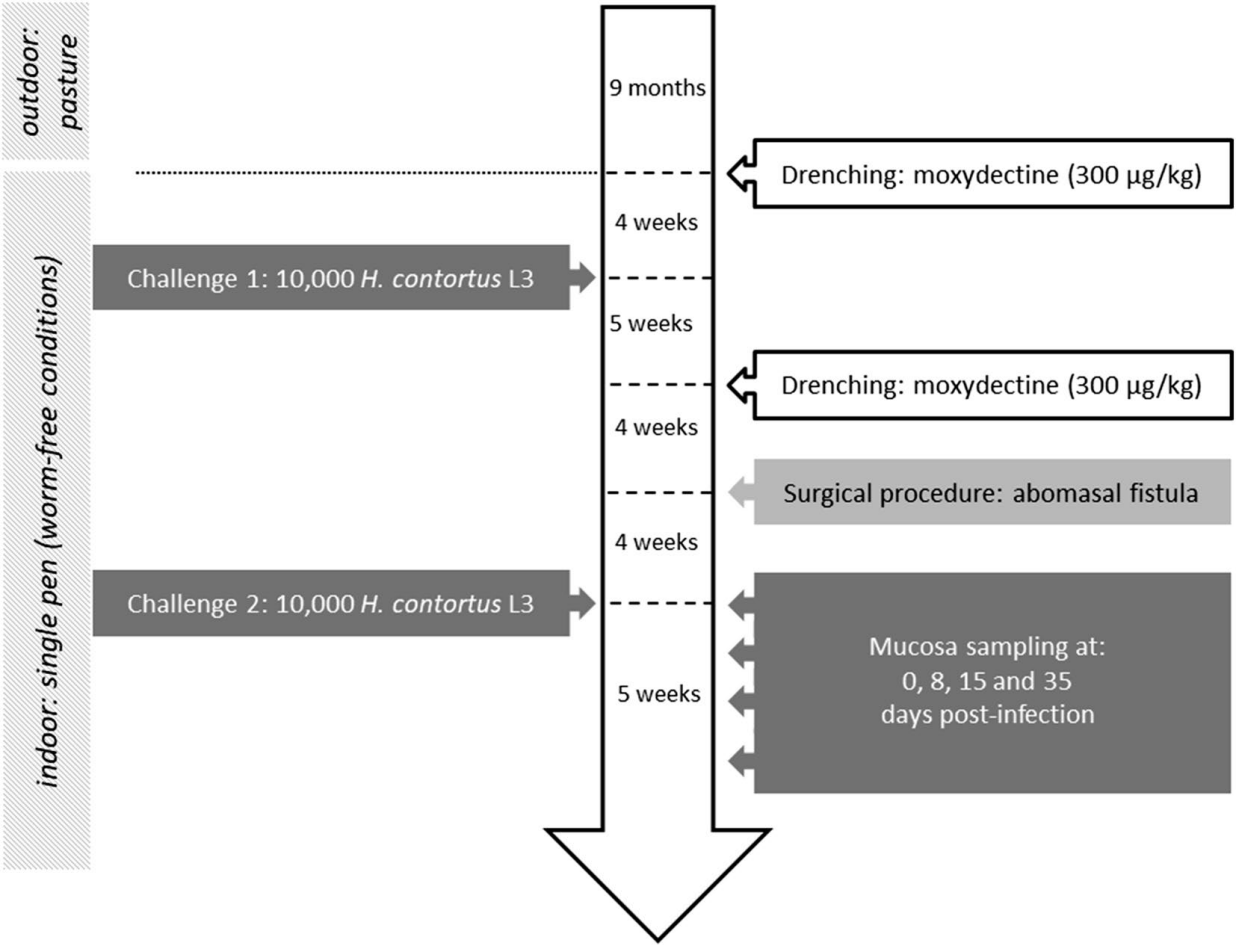
**Experimental design.** The Creole kids reared at pasture were chosen from the experimental flock at 9 month-old. The animals were drenched and housed indoors under worm-free conditions in a single pen. Kids were orally infected with a single dose of 10 000 *Haemonchus contortus* third-stage larvae (L3) (challenge 1 and 2, lasted for 5 weeks each).

The experimental design is described in Figure 1. Nine month-old Creole kids were chosen from the experimental flock of PTEA (Plateforme Tropicale d’Expérimentation sur l’Animal) in which the estimated breeding value (EBV) was calculated regularly since 1995 for each animal for FEC at 11 months of age following natural mixed infection on pasture taking into account the FEC of its ascendants and pedigree. Before the experiment the kids were reared at pasture with a limited level of GIN contamination (FEC < 500). The FEC of the 8 kids (*n* = 4 resistant and *n* = 4 susceptible), chosen on the basis of their extreme EBV in their cohort, were not statistically different. The EBV was estimated by taking into account the FEC of their ascendants and their pedigree. The averages EBV of the 2 groups were distant by 1.04 genetic standard deviation. The animals were drenched with moxidectine (Cydectine^®^, Fort Dodge Veterinaria S.A., Tours, France, 300 µg/kg) and housed indoors under worm-free conditions in a single pen, 1 month before the start of the experiment. Kids were orally infected with a single dose of 10 000 *Haemonchus contortus* third-stage larvae (L3) in two consecutive challenges. Each challenge lasted for 5 weeks with 8 weeks interval between the end of challenge 1 and the start of challenge 2. At the end of the challenge 1, the kids were drenched with moxidectin (Cydectine^®^, Fort Dodge Veterinaria S.A., Tours, France, 300 µg/kg) and 4 weeks later a fistula was surgically implanted in the abomasum of each animal to allow abomasal mucosa sampling at 0, 8, 15 and 35 dpi. After another period of 4 weeks, the animals were orally infected with a single dose of 10 000 *H. contortus* L3 (challenge 2). For FEC measurements during the experimental infection, approximately 10 g of faeces were collected in plastic tubes directly from the rectum of each animal, and transported from the experimental facility to the laboratory in refrigerated vials. The samples were individually analysed using a modified McMaster method for rapid determination and FEC was expressed as the number of eggs/g faeces [9].

### Surgical procedure

The custom designed abomasal cannula consisted of a flexible plastic tube with a length of 7 cm and a diameter of 2 cm with a rounded base of 4 cm in diameter. This flexible plastic was chosen to limit the possibility of mechanical abrasion of the mucosal surface of the abomasum. The animals were fasted 16 h before cannula insertion surgery. The animals were premedicated with ketamine (2 mg/kg IV, Le Vet Pharma, Wilgenweg, Netherlands), xylazine (0.2 mg mg/kg IM, Le Vet Pharma, Wilgenweg, Netherlands) and oxytetracycline (20 mg/kg IM, Eurovet Animal Health, Handelsweg, Netherlands). The animals were positioned in left lateral recumbency. Skin over the surgical site was shaved and prepped with povidone iodine (Vétédine, Laboratoire Vetoquinol S.A., Lure, France). A ventral midline incision was made to locate and externalise the abomasum. A 3 cm purse-string suture (Silk 2-0) was placed midway between the lesser and greater curvature and a stab incision was made in the center to insert the cannula. Then, the purse-string suture was tightened and tied off. To maintain the abomasum in an anatomically correct position, another stab incision was made in the abdominal wall at 10 cm from the laparotomy incision on the right paramedian area to enable the cannula to be passed freely through. An external flange was placed over the external part of cannula and fixed with adhesive fabric plaster strip. A sterile compress was inserted into the cannula as stopper. After the surgical procedure, all the animals were housed individually with free access to fresh water and hay.

### Biopsy sampling procedure

Biopsy specimens were taken from the abomasal mucosa using a flexible endoscope (FG-24 V, Pentax, France). The biopsies samples of 2 × 2 × 2 mm taken with the endoscopic forceps with window (model KW1815S) were quickly snap frozen into liquid nitrogen and stored at − 80 °C until RNA extraction. The animals were restrained in a harness made with a surgical drape allowing animal legs to protrude and which exposed the cannula. No sedation was used since no signs of discomfort or pain were observed during or after the procedure. The sterile compress inserted into the cannula was removed and the abomasal contents collected. The endoscope was introduced into the abomasal lumen and 3 biopsies per animal and per time points were taken from the abomasal folds of the fundic mucosa. At each time point the whole fundic mucosa was observed and no sign of mucosal injury due to the previous sampling was observed.

### RNA extraction and sequencing

Total RNA was extracted using the NucleoSpin^®^ RNA isolation kit (Macherey–Nagel, Hoerdt, France) following the manufacturer’s instructions, except that DNase digestion was performed with twice the indicated amount of enzyme. The total RNA concentration was measured with NanoDrop 2000 (ThermoScientific TM, France). The RNA integrity was verified using an Agilent Bioanalyzer 2100 (Agilent Technologies, France) with a RNA Integrity Number of > 7.5. The extracted total RNA was stored at − 80 °C until sequencing.

High-quality RNA from all samples was processed for the preparation of cDNA libraries using an Illumina TruSeq RNA sample prep kit for mRNA analysis following the Illumina’s protocols. After quality control and quantification, cDNA libraries were pooled in groups of 6 and sequenced on 5 lanes on the HiSeqTM 2000 (Illumina^®^ NEB, USA) to obtain approximatively 30 million reads (100 bp paired-end) for each sample with insert sizes ranging from 200 to 400 base pairs.

### Bioinformatics and data analysis

The quality control check on raw reads in FASTQ format were processed using FASTQC and the Q20, Q30 and GC contents of the clean data were calculated. The Salmon software (version 0.9.1) was used for transcript quantification [23]. NCBI RefSeq reference transcript of the *Capra hircus* genome (assembly ARS1) was used to build the index within Salmon. The reads from each sample were mapped to the same index and quantified. Unix commands were used to obtain corresponding gene and transcript identifiers from the NCBI RefSeq annotation of the *Capra hircus* (ARS1). Using these identifiers, the tximport (version 1.8.0) package was used to import data into the R software (v3.5.1) and summarize the TPM estimates obtained from the Salmon tool of all samples at the gene level [24]. This process produced a global count file on which the statistical analyses were performed. A threshold of greater than or equal to 5 counts across samples was applied in order to remove genes showing low expression.

Partial least squares discriminant analysis (PLSDA) had been conducted using the mixomics package within R [25]. In this analysis, x was the matrix of gene expression values (count table) and the classes of y were given as resistant and susceptible. Each row of the x matrix represented the gene expression values for a sample, and each column corresponded to a gene.

Differentially expressed genes (DEG) of read counts were identified using the Bioconductor package DESeq2 within R [26]. Ten comparisons were performed; three comparing day 0 with day 8, 15 or 35 post-infection in the susceptible group, another three comparing the same days in the resistant group and four comparing samples from resistant versus susceptible animals at day 0, 8, 15 and 35 post-infection. To account for multiple testing, genes were filtered using a Benjamini and Hochberg false discovery rate (FDR) of < 0.001. Final DEG were determined on the basis of their fold change values to be log_2_ ≥ 1.0 for up-regulated genes and ≤ − 1.0 for down-regulated genes. Gene ontology (GO) analysis for the biological processes was performed to identify the biological function classification of the genes, which describes properties of genes and their products. DEG are functionally grouped into the biological processes looking for significantly enriched functions compared to the human genomic background due to the lack of goat (*C. hircus*) GO data. GO enrichment analysis and GO annotations plotting were performed using the clusterProfiler R package [27]. All enriched GO terms that possessed a *p*-value < 0.01 were displayed and the top 5 biological processes for each comparison were plotted. Analysis of canonical pathways and regulator effects were performed using Ingenuity pathway analysis (IPA) software (Ingenuity Systems, Redwood City, CA, USA) for DEG in each comparison.

Faecal egg counts (FEC) were measured twice a week after infection from 21 to 36 dpi. The FEC variance was normalized using log transformation. PROC MIXED procedure (v. 9.4, SAS Inst. Inc., Cary, NC, USA, 2012) was used to test statistical differences. The differences were considered significant when *p* < 0.05. The results are presented after back transformed.

### Quantitative real-time PCR (qRT-PCR) validation

To validate the results of the RNAseq analysis, the gene expression for a total of 9 genes (*n* = 6 for each comparison: resistant vs susceptible at 0, 15 and 35 dpi, and resistant and susceptible for 0 vs 8 dpi, 0 vs 15 dpi and 0 vs 35 dpi) was determined by qRT-PCR. The endogenous control for all reactions was goat *ACTB* (*actin beta*) gene whose expression remained stable among the samples. The cDNA was synthetize with a total of 2 µg of high quality total RNA (RIN > 7.5) by using M-MLV Reverse Transcriptase (Promega, Charbonières, France) according to the manufacturer’s instructions. All qRT-PCR reactions were carried out in 48-well plates in a Prime Pro 48 Real-Time PCR System and analyzed with the ProStudy Software v5.2.10 (Techne, Staffordshire, UK). Taqman^®^ predesigned gene expression assay (Table 1) and the universal PCR master mix were purchased from Applied Biosystems and the analysis were performed according to the manufacturer’s instructions (ThermoFisher Scientific, Applied Biosystems, Courtaboeuf, Villebon-sur-Yvette, France). Samples were analyzed in duplicate in a total volume of 20 μL containing: 4 μL of cDNA, 10 μL of 2X TaqMan^®^ Fast Advanced Master Mix, 1 µL of TaqMan^®^ Gene Expression Assays 20X (ThermoFisher Scientific, Applied Biosystems, Courtaboeuf, France) and 5 μL of distilled RNAse DNAse-free water. Relative gene expression values were determined using relative quantification (2^−ΔΔCt^ method, [28]).

**Table 1:** **List of target genes for qRT-PCR validation and assay IDs according to the manufacturer**

Gene symbol	Gene description	Assay IDs
*ACTB*	*actin beta*	Ch04810274_s1
*CYP4F2*	*phylloquinone omega*-*hydroxylase*	Ch04672252_m1
*DUOXA2*	*dual oxidase maturation factor 2*	Ch04786286_m1
*CCL20*	*C*–*C motif chemokine ligand 20*	Ch04791475_m1
*IFI6*	*interferon alpha inducible protein 6*	Ch04807049_g1
*LST1*	*leukocyte specific transcript 1*	Ch04741898_m1
*NKX6*-*3*	*NK6 homeobox 3*	Ch04677616_m1
*OLFM4*	*olfactomedin 4*	Ch04796577_m1
*TFF3*	*trefoil factor 3*	Ch04767901_m1
*TLR4*	*toll*-*like receptor 4*	Ch04654181_m1

## Results

### Parasitological measures

**Figure 2:**
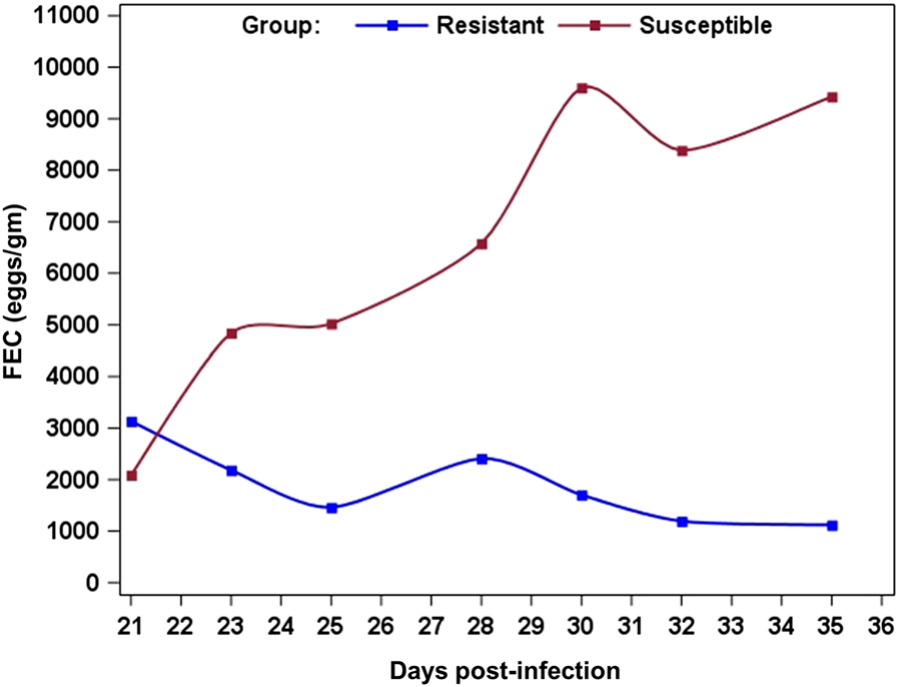
**Geometric means of fecal egg count (FEC) comparing resistant and susceptible animals.** Blue: resistant, Red: susceptible. The animals were experimentally infected with 10 000 *H. contortus* infective larvae (L3) at day 0 post-infection.

A significant effect of the group (i.e. resistant vs susceptible), the dpi and their interaction (*p* < 0.001) was observed for FEC (Figure 2). At 21 dpi no difference was observed between groups. Thereafter the FEC was significantly lower in resistant compared to susceptible animals whatever the dpi.

### RNA sequencing and variance analysis

**Figure 3:**
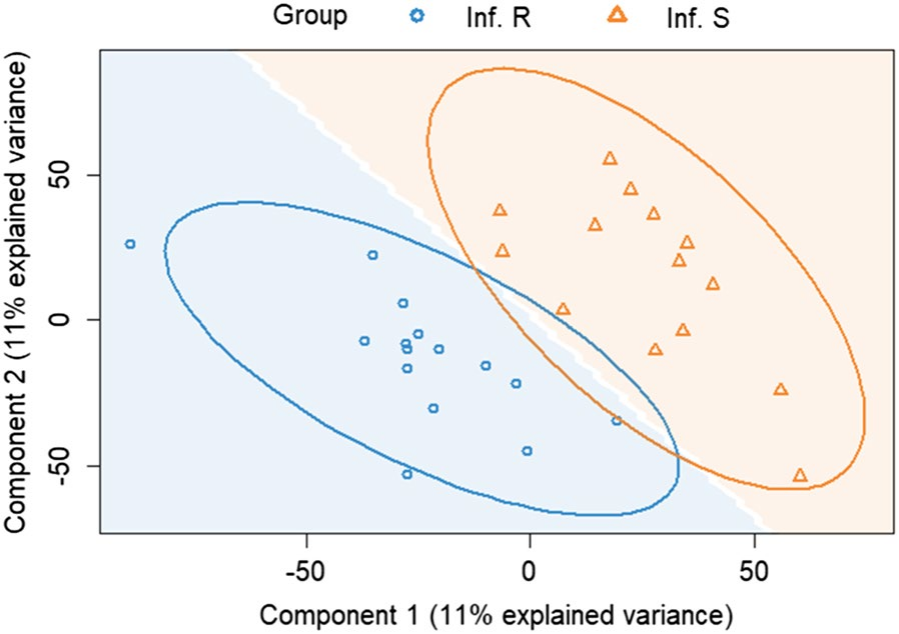
**Multilevel PLS-DA of the gene expression of infected resistant and susceptible animals.** Infected Resistant: Inf. R, Infected susceptible: Inf. S.

Alignment of RNA sequencing to the reference *Capra hircus* genome (assembly ARS1) resulted in an average of 4.5 ± 0.1 million reads per sample. These reads correspond to 23 258 genes of the goat genome. A total of 15 188 out of the 23 258 annotated genes (65%), showed at least 5 read counts per row and were used in the subsequently analysis. The multilevel PLSDA for gene expression of infected resistant and susceptible kids explained more than 20% of the variance in its two-dimension components (Figure 3). Component 1 represented 11% of the whole variability and component 2 represented also 11% of the variation.

### Differential gene expression

The numbers of DEG for each comparison are shown in Table 2. The numbers of DEG were low for the comparison between groups (R vs S) whatever the time point. For the comparison within infected resistant or infected susceptible, the numbers of DEG were lower for 0 versus 15 dpi (678 and 1748, respectively) compared with 0 versus 8 or 0 versus 35 dpi. Meanwhile the highest number for DEG was recorded for the comparison of 0 versus 35 dpi of infected susceptible (3316) and infected resistant (2263). The fold change was on average higher when comparing different time points within each group (from − 11.15 to 24.17 and from − 11.83 to 9.30 for R or S respectively) than between groups at different days. Human orthologues were mapped for 72–85% of the DEG (Table 2).

**Table 2:** **Number of differentially expressed genes (n) for the different comparisons including log**_**2**_**fold change and the number of human orthologues (including proportion of genes with human orthologues)**

Comparison	n	Log_2_ fold change	Human orthologues
Inf. R 0 vs 8 dpi	1336	− 11.15, 24.17	1017 (76.12%)
Inf. R 0 vs 15 dpi	678	− 10.81, 4.57	549 (80.97%)
Inf. R 0 vs 35 dpi	2263	− 10.58, 6.66	1881 (83.12%)
Inf. S 0 vs 8 dpi	2221	− 10.60, 9.30	1744 (78.52%)
Inf. S 0 vs 15 dpi	1748	− 11.84, 8.82	1439 (82.32%)
Inf. S 0 vs 35 dpi	3316	− 11.83, 9.23	2811 (84.77%)
R vs S 0 dpi	456	− 7.39, 6.00	337 (73.90)
R vs S 8 dpi	679	− 4.27, 27.7	490 (72.16%)
R vs S 15 dpi	318	− 5.1, 7.82	247 (77.67%)
R vs S 35 dpi	758	− 7.34, 8.48	579 (76.39%)

*Inf. R* infected resistant, *Inf. S* infected susceptible, *R vs S* resistant versus susceptible, *dpi* days post-infection.

− Log_10_ p-value: − Log_10_ (p-value).

### Validation of expression by qRT-PCR

**Figure 4:**
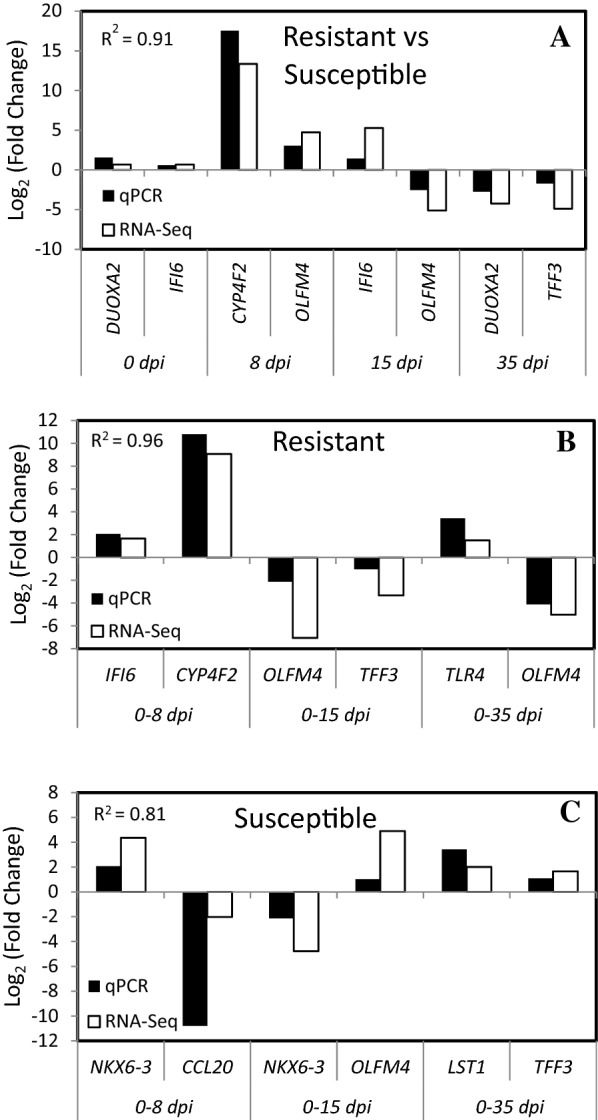
**Fold change of differentially expressed genes measured by RNA-Seq (white) and qRT-PCR analyses (black)**. RNA-Seq: white bars, qRT-PCR: black bars. The results are presented according to the comparisons: resistant versus susceptible at 0, 8, 15 and 35 days post-infection (dpi), 0 versus 8, 15 and 35 dpi for resistant and susceptible animals respectively.

qRT-PCR for nine genes was performed to validate RNA sequencing results. For the comparison of resistant versus susceptible animals at 0, 8, 15 and 35 dpi, the genes selected randomly among the DEG were: *DUOXA2*, *IFI6*, *CYP4F2*, *OLFM4* and *TFF3*. For the comparison of 0 versus 8, 15 and 35 dpi within the resistant and the susceptible animals the genes were respectively: IFI6, CYP4F2, OLFM4, TFF3, TLR4 and NKX6-3, CCL20, OLFM4, LST1, TFF3. The log_2_ fold change levels of the selected genes measured by qRT-PCR were in good agreement with the values from the sequencing data (Figure 4). The gene expression patterns from qRT-PCR were highly correlated with the sequencing results: the correlation coefficients were respectively 0.91, 0.96 and 0.81 for the comparison of resistant versus susceptible animals at different time points and the comparison of 0 versus other time points within the resistant and the susceptible animals.

### Functional classification analysis

#### Gene ontology (GO)

**Figure 5:**
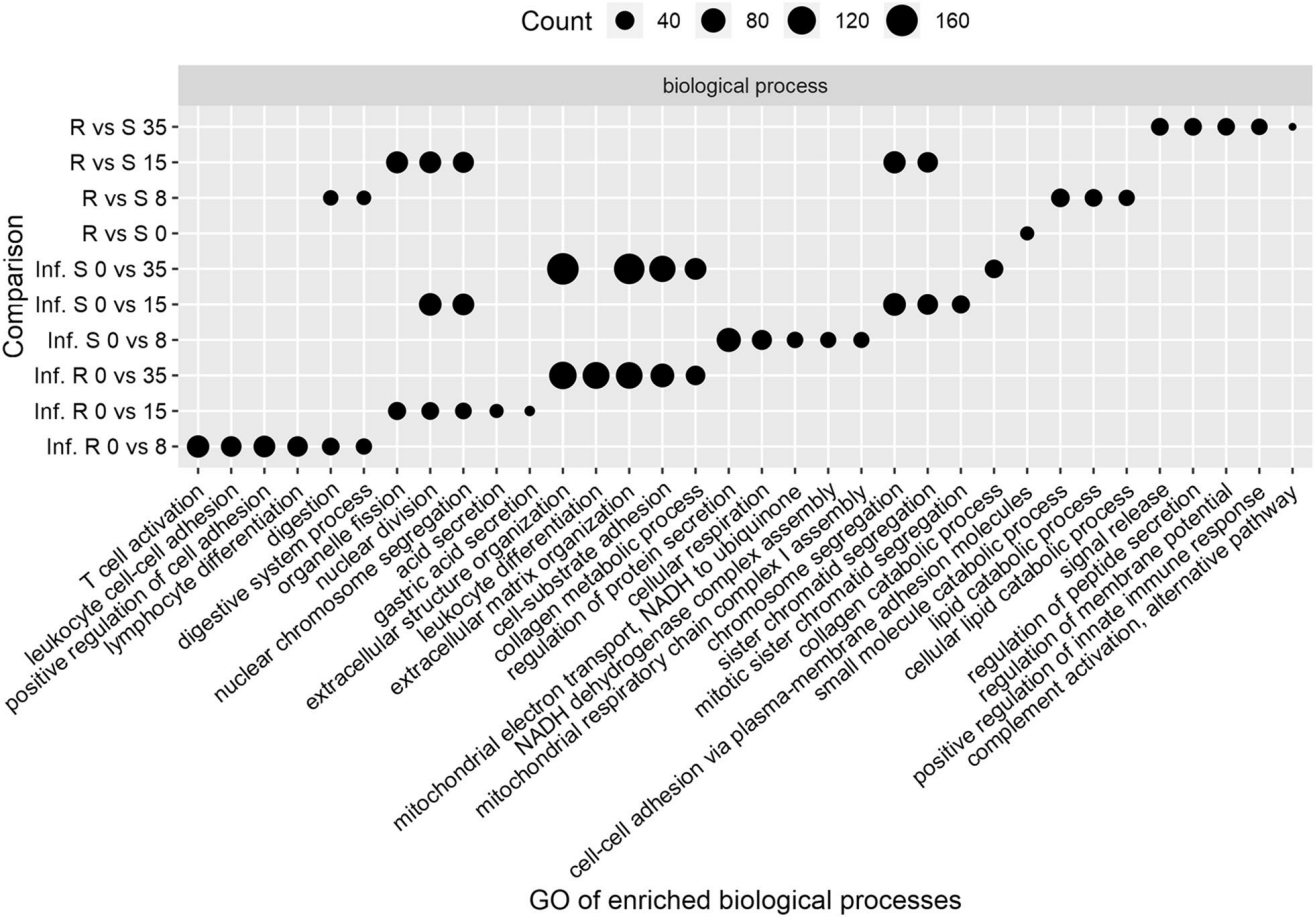
**Gene Ontology (GO) of the top 5 biological processes for the three comparisons.** Infected resistant: Inf. R, infected susceptible: Inf. S and resistant versus susceptible (R vs S) animals. iR vs S: non-infected Resistant compared to Susceptible animals. Inf. S: Infected Susceptible animals (comparison between days post-infection within the susceptible animals). Inf. R: Infected Resistant animals (comparison between days post-infection within the resistant animals). 0, 8, 15 and 35: days post-infection.

An enriched GO term analysis for biological processes was performed using the DEG from each comparison. The top 5 significant biological processes in each term are presented in Figure 5. Comparing 0 versus 35 dpi, four out of the top 5 biological processes were the same for the resistant and the susceptible kids; meanwhile leukocyte differentiation was in the top biological process only for the resistant kids. The comparison of infected resistant at 0 versus 8 dpi showed biological processes related to the immune response within the top 5 significant processes (e.g. T cell activation, leukocyte cell–cell adhesion and lymphocyte differentiation). Positive regulation of the innate immune response was in the top 5 biological processes when comparing susceptible with resistant at 35 dpi.

#### Pathway enrichment analysis

**Figure 6:**
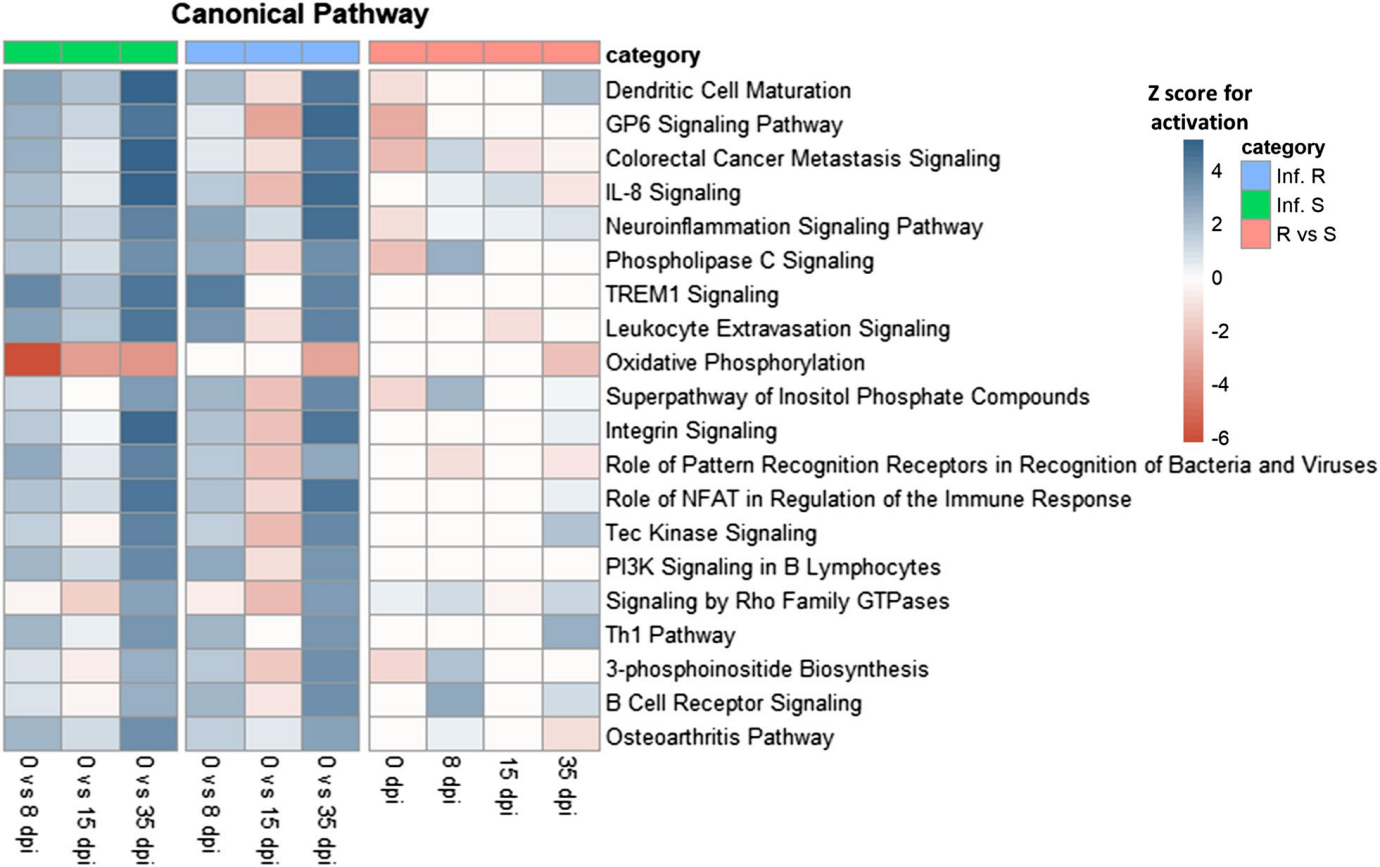
**Top 20 canonical pathways of differentially expressed genes for infected resistant and susceptible animals.** Infected Resistant: Inf. R, Infected susceptible: Inf. S. Comparison of day 0 with 8, 15 and 35 dpi and resistant versus susceptible animals (R vs S) at 0, 8, 15 and 35 dpi. The color gradient moves from red (down-regulation, z-score for activation = − 6) to blue (up-regulation, z-score for activation = four). Z-score for activation: according to Ingenuity systems, the activation z‐score is used to infer likely activation states of upstream regulators based on comparison with a model that assigns random regulation directions.

**Figure 7:**
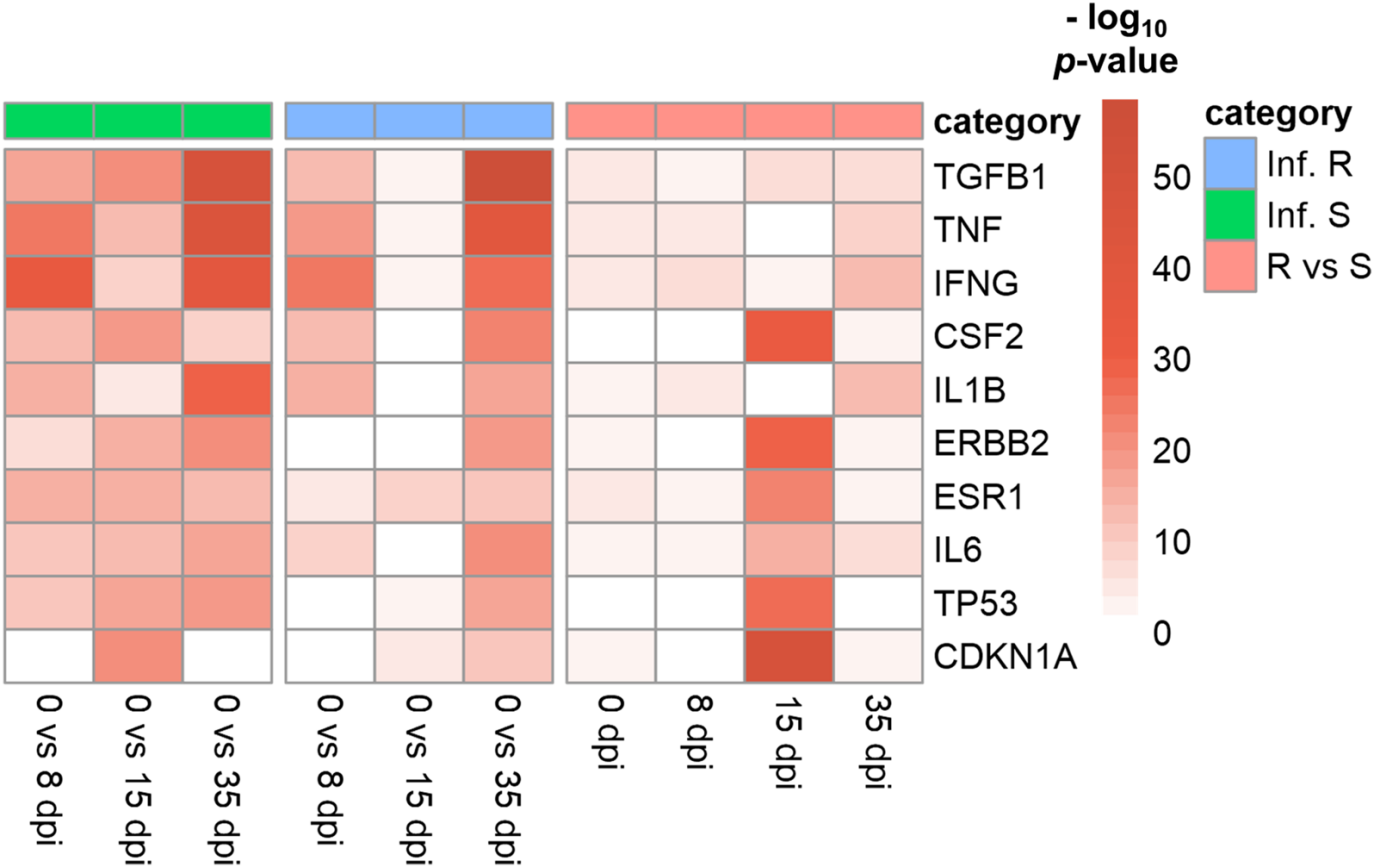
**Top 10 upstream regulators of differentially expressed genes for infected resistant and infected susceptible animals.** Infected Resistant: Inf. R, Infected susceptible: Inf. S. Comparison of day 0 with 8, 15 and 35 dpi and resistant versus susceptible animals (R vs S) at 8, 15 and 35 dpi. The color gradient moves from white [no significant difference, − Log_10_ (*p*-value) = 0] to red [significant difference, − Log_10_ (*p*-value) ≥ 2].

The Ingenuity Pathway Analysis was used to compare results from different comparison over time. The top canonical pathways (Figure 6) and the top upstream regulators (Figure 7) were compared. When comparing day 0 versus 35 post-infection, the top 20 canonical pathways showed a high activation of the immune response through dendritic cell maturation, *IL*-*8* signaling, Leukocyte extravasation signaling, *NFAT* in regulation of the immune response, *P13K* signaling in B lymphocytes, Th1 pathway and B cell receptor signaling pathways. In resistant compared with susceptible kids the B cell receptor signaling pathway was activated at 8 dpi while dendritic cell maturation and Th1 pathways were activated at 35 dpi.

The top 10 upstream regulators of the DEG for different comparisons showed that some genes like *TGF*-*β1*, *TNF*-*α*, *IFN*-*γ*, *IL1*-*β* and *IL*-*6* were in the group of the top significant upstream regulators in both infected resistant and susceptible kids specially when comparing 0 versus 35 dpi. These genes were still significantly differently expressed between resistant and susceptible kids at 35 dpi (Figure 7). The *TGF*-*β1* gene was the top significant upstream regulator that was differently expressed in resistant compared with susceptible kids in the abomasal mucosa.

### Differential of CD4+ T cell

**Figure 8:**
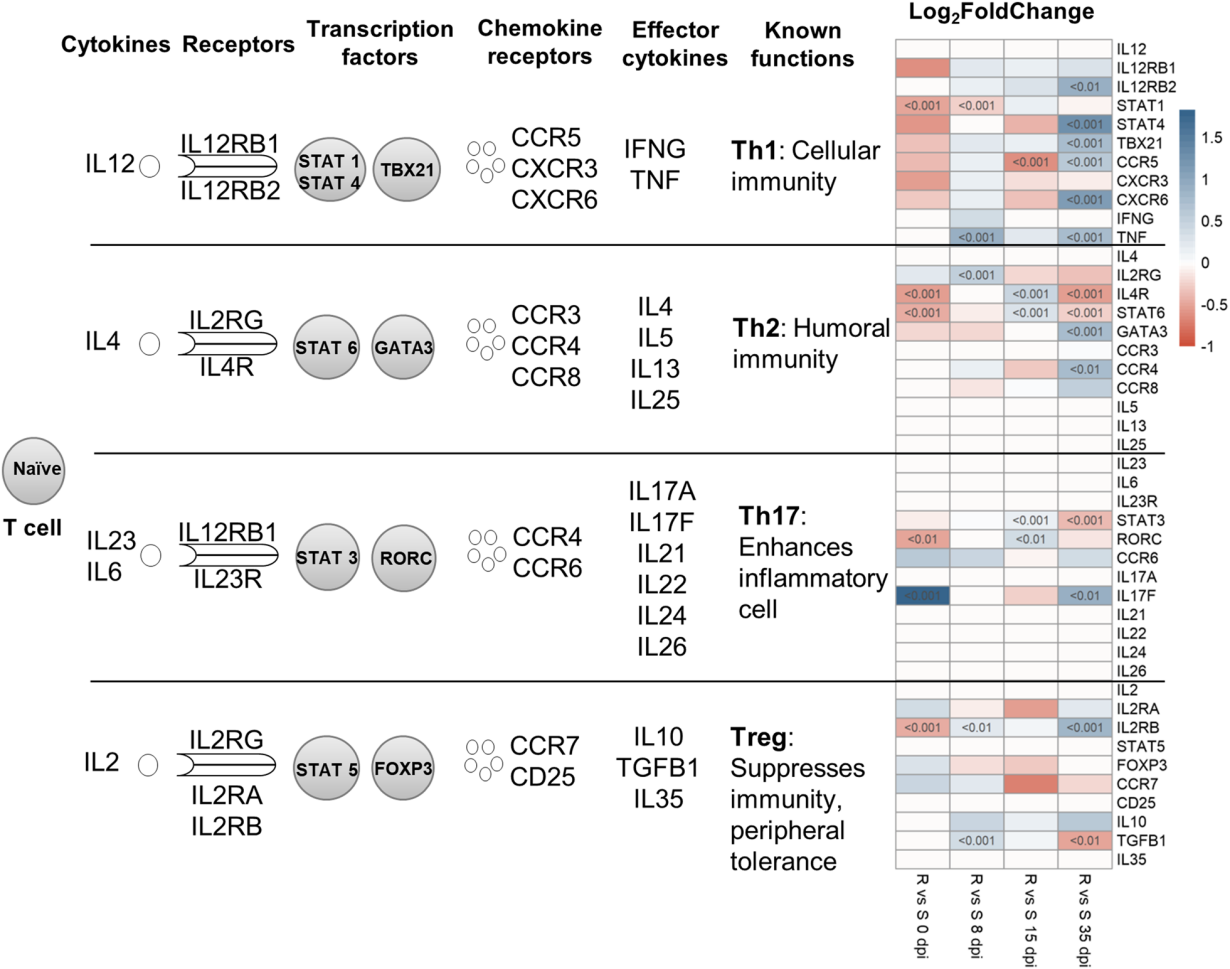
**Differential of CD4+ T cell activation and gene expression controlling the differences between resistant and susceptible animals.** Comparison between resistant and susceptible animals (R vs S) at 0, 8, 15 and 35 dpi. The color gradient moves from red (Log_2_ Fold Change range from − 1 to 0) to blue (Log_2_ Fold Change range from 0 to 1.5).

Genes related to the CD4+ T cell activation and the fold change comparing resistant versus susceptible kids at 0, 8, 15 and 35 dpi are presented in Figure 8. The CD4+ T cell differentiation pathway showed a significant difference and a positive fold change for the majority of genes controlling the Th1 pathway when comparing resistant versus susceptible kids at 35 dpi. The expression of genes controlling the Th2 pathway showed time series activation in resistant compared with susceptible kids at different dpi: *IL2RG* activated at 8 dpi, *IL4R* and *STAT6* at 15 dpi, *GATA3* and *CCR4* at 35 dpi. Meanwhile the expression of *IL4R* and *STAT6* at 35 dpi is higher in susceptible kids. The expression levels for genes controlling the Th17 pathway showed a positive fold change for *STAT3* and *RORC* in resistant kids at 15 dpi, then for *IL17F* at 35 dpi while for *STAT3* the expression was higher in susceptible kids at 35 dpi. Comparing resistant versus susceptible at 0 dpi (before the experimental infection), the expression of *IL17F* was three times higher in resistant kids. No difference of *FOXP*-*3* expression was observed between resistant and susceptible whatever the dpi, while the expression of *TGF*-*β1* was significantly higher in resistant kids at 8 dpi and lower at 35 dpi.

## Discussion

This study aimed to investigate the kinetic changes in mucosal molecular pathways and immunity development of resistant and susceptible Creole kid goats in response to *H. contortus*. The classification of the animals as resistant or susceptible was explained at 22% by the gene expression profile. *H. contortus* infection induced a high number of DEG in the mucosa of both resistant and susceptible animals whatever the time points while the numbers of DEG were much lower when comparing resistant versus susceptible animals at the different time points of infection. This result indicates that most genes involve in the host response against *H. contortus* infection were similar in susceptible and resistant animals.

GO of enriched biological processes showed an earlier activation of immune biological processes in resistant kids. Indeed, the top biological processes at 8 dpi were T cell activation, leukocyte cell–cell adhesion and lymphocyte differentiation. One of the top significant pathways was B cell receptor signaling. In keeping with this results, McRae et al. reported an early immune response to *Teladorsagia circumcincta* in resistant sheep at 7 dpi [19]. The same top four biological processes were observed in resistant and susceptible animals when comparing 0 and 35 dpi. However, none of these processes appeared in these top biological processes when comparing susceptible with resistant animals at 35 dpi, suggesting that at 35 dpi the host priority at the abomasal mucosa interface would be similar for resistant and susceptible kids.

The Th1 pathway was one of the top pathways identified in most of the comparison performed in this study. Upstream regulators of the genes involved in the Th1 processes include *TNF*-*α* and *IFN*-*γ*, which were also identified as DEG. In accordance with this result, a transient increase of the expression of *TNF*-*α* and *IFN*-*γ* was observed earlier after *H. contortus* infection in sheep both in the abomasal mucosa and the draining lymph nodes [29–31]. However, a non-protective Th1 response associated with an increased expression of cytokines, as *TNF*-*α* and *IFN*-*γ*, was observed respectively in susceptible and primary infected sheep infected with *H. contortus* [32, 33]. Indeed, studies on murine models demonstrated for a long time that the protective response against GIN parasites is better associated with the Th2 polarization of the immune response [34], while host susceptibility is associated with a Th1 response [35, 36]. In ruminants, the Th1/Th2 dichotomy remains controversial despite studies showing a correlation between host resistance and a polarized Th2 immune response [37–39]. A simultaneous increased expression of Th1- and Th2-type cytokines was shown in cattle infected with *Ostertagia ostertagi* [40–42]. Similarly, looking at differential of CD4+ T cell, we found signals for Th1 and Th2 activation at 35 dpi in resistant animals when comparing them with susceptible animals. Caucheteux et al. [43] reported that the expression of IL1-β gives rise to inflammatory Th2 cells that are specialized to induce allergic inflammatory responses, whereas Th2 primed in the absence of IL1-β are more important as regulatory cells, that is amplifiers of Th2 cells and antibody response by B cells. Our results showed IL1-β in the top upstream regulator genes controlling infection response.

Transforming growth factor beta (TGF-β) is a multifunctional cytokine known for its regulatory activity and the induction of peripheral tolerance [44]. We found that the gene expression profile of TGF-*ß1* was the top significant upstream regulator when comparing the dynamics of infection in resistant and susceptible animals. TGF-*ß1* was activated in susceptible and inhibited in resistant animals at 35 dpi. The same was previously reported in other studies in goats [22, 45] and also a study on sheep infected with *H. contortus* [15]. The underlying mechanisms could be a manipulation of the host immune response by *H. contortus*, notably through the induction of the secretion of *IL*-*10* and *TGF*-*ß1* by goat monocytes to promote an anti-inflammatory environment favorable for worm survival [46]. This hypothesis needs to be investigated.

A gene expression profiling study of the abomasal mucosal and lymph nodes of resistant and susceptible goats in response to *H. contortus* infection at 42 dpi has previously reported that the maintenance of the integrity of the mucosal barrier is one of the priorities of the host response at the late stage of infection [22]. The study presented here studied the dynamics of the gene expression in the goat abomasal mucosa in response to *H. contortus* infection using information from the whole transcriptome of resistant and susceptible kids. A time series activation of Th2 genes was identified for resistant animals compared with the susceptible ones. The later activation of some genes in susceptible animals indicated that the Th2 response was activated earlier in resistant kids compared to susceptible kids. Transcriptional profiling of the abomasal lymph node from Scottish Blackface lambs showed that resistant animals are generating an earlier immune response to *T. circumcincta* infection compared to susceptible animals [19]. This difference was through pathways relating to the inflammatory response, migration of T lymphocytes and synthesis of reactive oxygen species [19].

IL17 is the leading inflammatory cytokine in the Th17 cell populations [47]. Neither the IL17A nor the IL17F genes have been described in studies analyzing the resistance to GIN in sheep. Nonetheless, IL17 transcripts have been shown to be upregulated in the bovine abomasal mucosa after 24 days of single *O. ostertagi* challenge and 60 days of trickle experimental or natural infection [42]. However, the positions of these interleukin genes have been found to be relatively close to the DRB1 gene in sheep [48], which has been reported to be associated with GIN resistance in sheep [49, 50]. In the present study, *IL17F* was the gene showing the most significant expression difference at day 0 of infection, having an expression three times higher in resistant compared with susceptible kids. Future experiments should investigate the potential of this gene as a pertinent biomarker in a selection program.

The present study showed that *H. contortus* infection in goat induces a marked immune response at the mucosal level in resistant animals, which is characterized by the simultaneous upregulation of Th1 and Th2 genes. Our results suggested differences in the time series activation for Th2 genes, indicating that the immune response is activated earlier in resistant kid goats compared to the susceptible ones. We also found that TGF-*ß1* has a major regulator role during GIN infection in goats.

## Data Availability

All data generated during this study are available in the NCBI SRA repository. All other relevant data are included in this published article.
